# A novel *RPE65* variant p.(Ala391Asp) in Leber congenital amaurosis: a case report and literature review in Japan

**DOI:** 10.3389/fmed.2024.1442107

**Published:** 2024-09-18

**Authors:** Natsuki Higa, Takaaki Hayashi, Kei Mizobuchi, Maki Iwasa, Shingo Kubota, Kazuki Kuniyoshi, Shuhei Kameya, Hiroyuki Kondo, Mineo Kondo, Tadashi Nakano

**Affiliations:** ^1^Department of Ophthalmology, Katsushika Medical Center, The Jikei University School of Medicine, Tokyo, Japan; ^2^Department of Ophthalmology, The Jikei University School of Medicine, Tokyo, Japan; ^3^Department of Ophthalmology, Shiga University of Medical Science, Otsu, Japan; ^4^Kubota Eye Clinic, Kusatsu, Shiga, Japan; ^5^Department of Ophthalmology, Kindai University Faculty of Medicine, Osaka-sayama, Japan; ^6^Kameya Eye Clinic, Chiba, Japan; ^7^Department of Ophthalmology, University of Occupational and Environmental Health, Fukuoka, Japan; ^8^Department of Ophthalmology, Mie University Graduate School of Medicine, Tsu City, Japan

**Keywords:** inherited retinal dystrophy, Leber congenital amaurosis, electroretinography, RPE65 gene mutation, whole exome sequencing

## Abstract

**Introduction:**

In Japan, inherited retinal dystrophy caused by biallelic variants of the *RPE65* gene is exceedingly rare. The purpose of this study was to describe a Japanese male patient with a novel variant in *RPE65* associated with Leber congenital amaurosis (LCA).

**Case report:**

The patient, diagnosed with LCA, exhibited infantile nystagmus and reported experiencing night blindness since early childhood. At 27 years of age, the patient underwent an ophthalmologically evaluation. Corrected visual acuity was Snellen equivalent 20/133 in the right eye and Snellen equivalent 20/100 in the left eye. Fundus examination revealed alterations in the retinal pigment epithelium characterized by hypopigmentation and narrowing of retinal vessels. Fundus autofluorescence imaging demonstrated a generally diminished autofluorescent signal. Full-field electroretinography identified a generalized dysfunction of both rod and cone systems in each eye. Whole exome sequencing identified a novel missense variant in *RPE65* (NM_000329.3): c.1172C > A p.(Ala391Asp), which was classified as pathogenic, as well as a recurrent variant p.(Arg515Trp).

**Conclusion:**

This study provides valuable insights into the genotype–phenotype correlation of *RPE65*-associated LCA in Japanese patients, with critical implications for enhanced diagnostic accuracy and informed therapeutic decisions.

## 1 Introduction

Inherited retinal dystrophies (IRDs) constitute a diverse group of genetically and clinically heterogeneous inherited retinal disorders characterized by progressive retinal degeneration that culminates in legal blindness. A particularly severe form of IRDs, Leber congenital amaurosis (LCA), manifests with profound congenital or early-onset vision loss and nystagmus (1). Previous reports have indicated that pathogenic variants in genes such as *CEP290, GUCY2D, CRB1, RDH12*, and *RPE65* are predominantly associated with LCA and early-onset severe retinal dystrophy (EOSRD) (1, 2).

The *RPE65* gene (NM_000329.3) is located on the short arm of chromosome 1 (1p31.3) and spans 14 exons over 21.1 kilobase pairs. The encoded RPE65 protein, composed of 533 amino acids (NP_000320.1), functions within the retinal pigment epithelium, catalyzing the conversion of all-*trans*-retinyl fatty acid esters into 11-*cis*-retinol, a crucial step in visual chromophore regeneration (1). Biallelic pathogenic variants in *RPE65* were first identified in LCA/EOSRD patients in 1997 (3, 4). Such variants in *RPE65* account for approximately 11.4% of cases in early-onset retinal dystrophies in Western populations (5). These variants are also implicated in autosomal recessive retinitis pigmentosa (RP) (6). However, *RPE65*-associated retinal dystrophies are reported to be relatively rare in Japan (7, 8), with few documented cases detailing their clinical progression (9–11).

In 2018, the FDA approved Voretigene neparvovec (Luxturna^®^) as the inaugural gene therapy for LCA/EOSRD with biallelic *RPE65* variants, following positive results from phase 3 clinical trial (12). Subsequently, Voretigene neparvovec was approved in Japan in 2023, post phase 3 clinical trials (NCT04516369). Despite the therapeutic potential of gene therapy, its implementation in Japan faces challenges. First, the progression of *RPE65*-LCA/EOSRD in Japanese patients is poorly documented. Additionally, the incidence of such dystrophies in Japan is considerably lower than reported in other populations. The second challenge pertains to the exceptionally low incidence rates among previously published reports on IRDs (7, 8). This report introduces a case of LCA in a young Japanese patient with biallelic *RPE65* variants and reviews the literature on Japanese patients with biallelic variants in *RPE65*.

## 2 Case report

A male patient, identified as JU2139, exhibited infantile nystagmus noticed by his parents before he was 1 year old. He has reported experiencing night blindness since early childhood, although no systemic diseases have been diagnosed. There was no history of consanguinity among his unaffected parents. The patient recalled having relatively good visual acuity upon entering elementary school. His first ophthalmological consultation occurred at 13 years of age at a local clinic, where he was observed for 3 years. Initial fundus photography indicated mild coarsening of the retinal pigment epithelium (RPE) bilaterally (OU). The patient was eventually diagnosed with LCA. By the age of 24, he achieved a decimal best-corrected visual acuity (BCVA) of 0.5 (Snellen equivalent 20/40) in the right eye (OD) and 0.4 (Snellen equivalent 20/50) in the left eye (OS).

**Figure 1:**
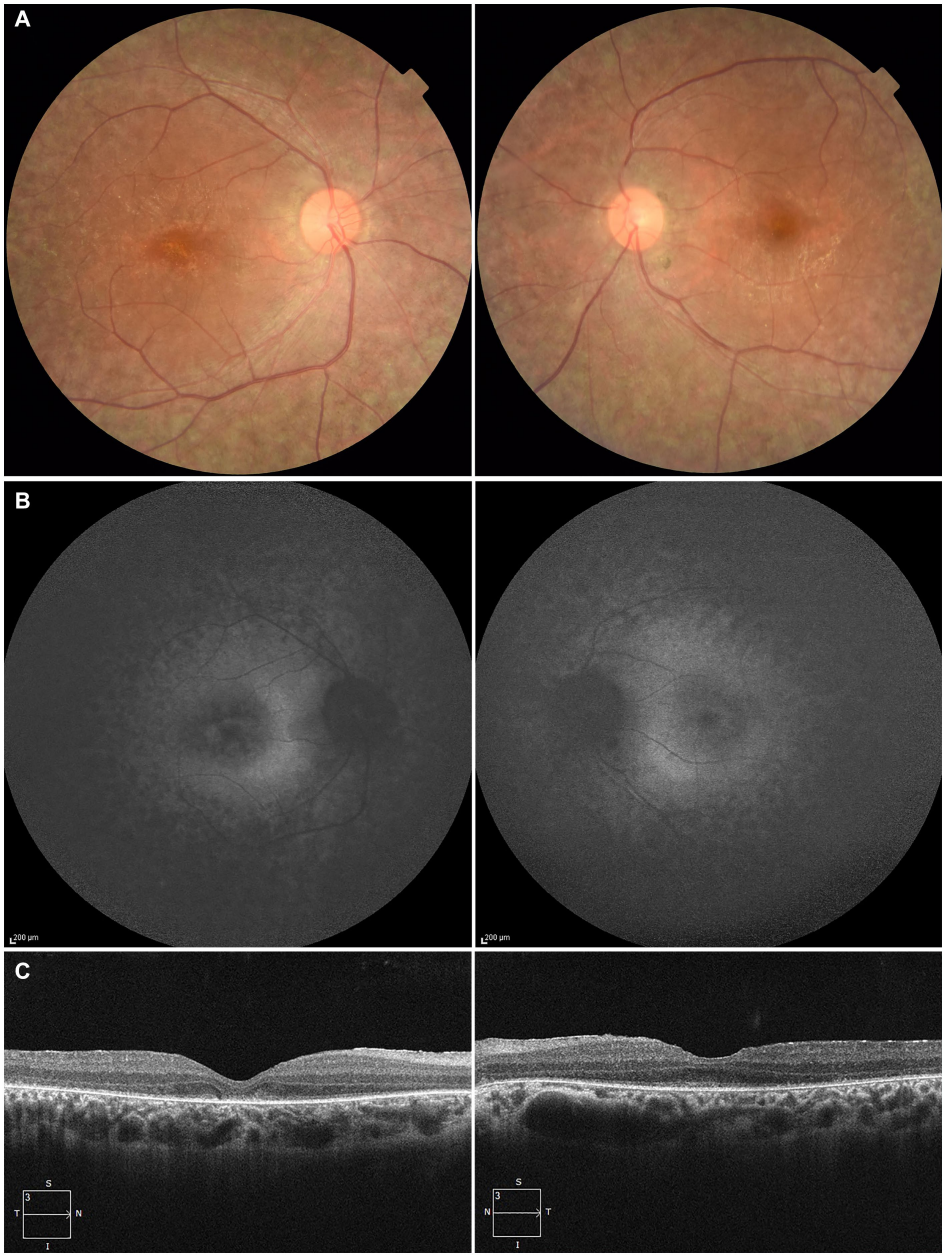
Multimodal imaging of the right eye (left column) and the left eye (right column) of the patient at the age of 27. **(A)** Fundus photography shows alterations in the retinal pigment epithelium characterized by hypopigmentation and narrowing of retinal vessels in both eyes. **(B)** Fundus autofluorescence imaging reveals areas of hyper-autofluorescence corresponding to the lesions and a generally diminished autofluorescent signal within and beyond the vascular arcades. **(C)** Optical coherence tomography reveals disruption of ellipsoid zone (EZ), interdigitation zone and external limiting membrane in the right eye. In contrast, the left eye exhibited a faintly preserved EZ in the foveal region.

**Figure 2:**
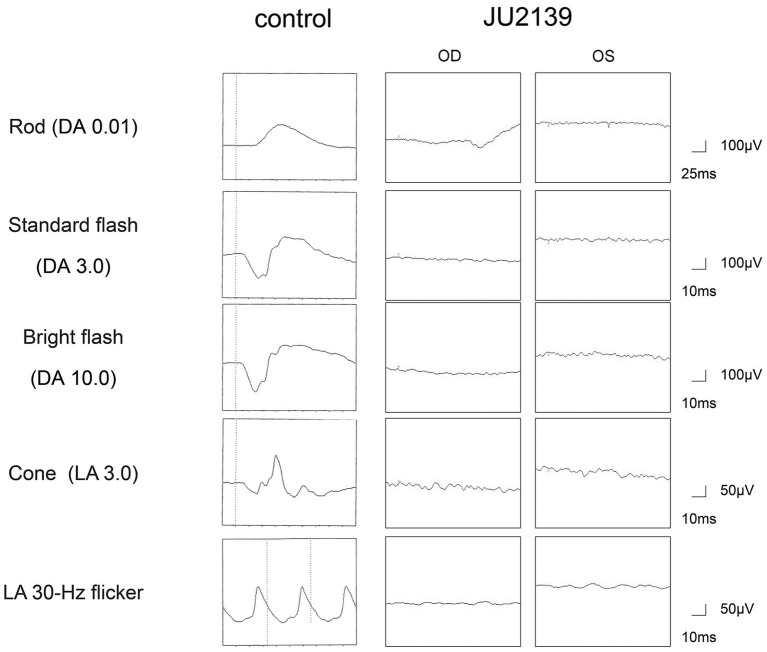
Full-field electroretinograms in an age-matched control and the patient (JU2139). The electroretinograms are recorded under both dark-adapted (DA) and light-adapted (LA) conditions in the right eye (OD) and in the left eye (OS). The recorded amplitudes of the rod (DA 0.01 cd s m^−2^) b-waves, standard/bright-flash (DA 3.0/10.0 cd s m^−2^) a- and b-waves, cone (LA 3.0 cd s m^−2^) a- and b-waves and LA 30-Hz flicker responses are undetectable in the patient, signifying a generalized dysfunction of both rod and cone systems.

At 27 years of age, the patient was referred to the Department of Ophthalmology at The Jikei University Hospital for evaluation of a suspected inherited retinal dystrophy. The examination revealed a decimal BCVA of 0.1 (Snellen equivalent 20/200) OD and 0.2 (Snellen equivalent 20/100) OS, with respective refractive corrections of +2.50 diopters sphere (DS) with −2.00 diopters cylinder (DC) at 180 degrees OD and +1.75 DS with −3.00 DC at 175 degrees OS. Intraocular pressure was measured at 17 mmHg OD and 14 mmHg OS. Slit-lamp examination of the anterior segment showed no remarkable abnormalities. Examination of the posterior segment through fundus photography disclosed alterations in the RPE characterized by hypopigmentation, as well as narrowing of retinal vessels OU (Figure 1A). Both conventional and ultra-widefield fundus autofluorescence (FAF) imaging revealed areas of hyper-autofluorescence corresponding to the lesions and a generally diminished FAF signal within and beyond the vascular arcades (Figure 1B). Optical coherence tomography (OCT) performed using a Cirrus 5000 system (Carl Zeiss Meditec AG, Dublin, CA, United States), revealed disruption of ellipsoid zone (EZ), interdigitation zone and external limiting membrane OD. In contrast, the left eye exhibited a faintly preserved EZ in the foveal region (Figure 1C). Full-field electroretinograms (ERGs) were obtained under both dark-adapted (DA) and light-adapted (LA) conditions with dilated pupils, induced by a combination of tropicamide and phenylephrine hydrochloride eye drops, in accordance with the International Society for Clinical Electrophysiology of Vision protocols (13). In the ERGs, the recorded amplitudes of the rod (DA 0.01 cd s m^−2^) b-waves, standard/bright-flash (DA 3.0/10.0 cd s m^−2^) a- and b-waves, cone (LA 3.0 cd s m^−2^) a- and b-waves and LA 30-Hz flicker responses were undetectable (Figure 2), signifying a generalized dysfunction of both rod and cone systems.

**Figure 3:**
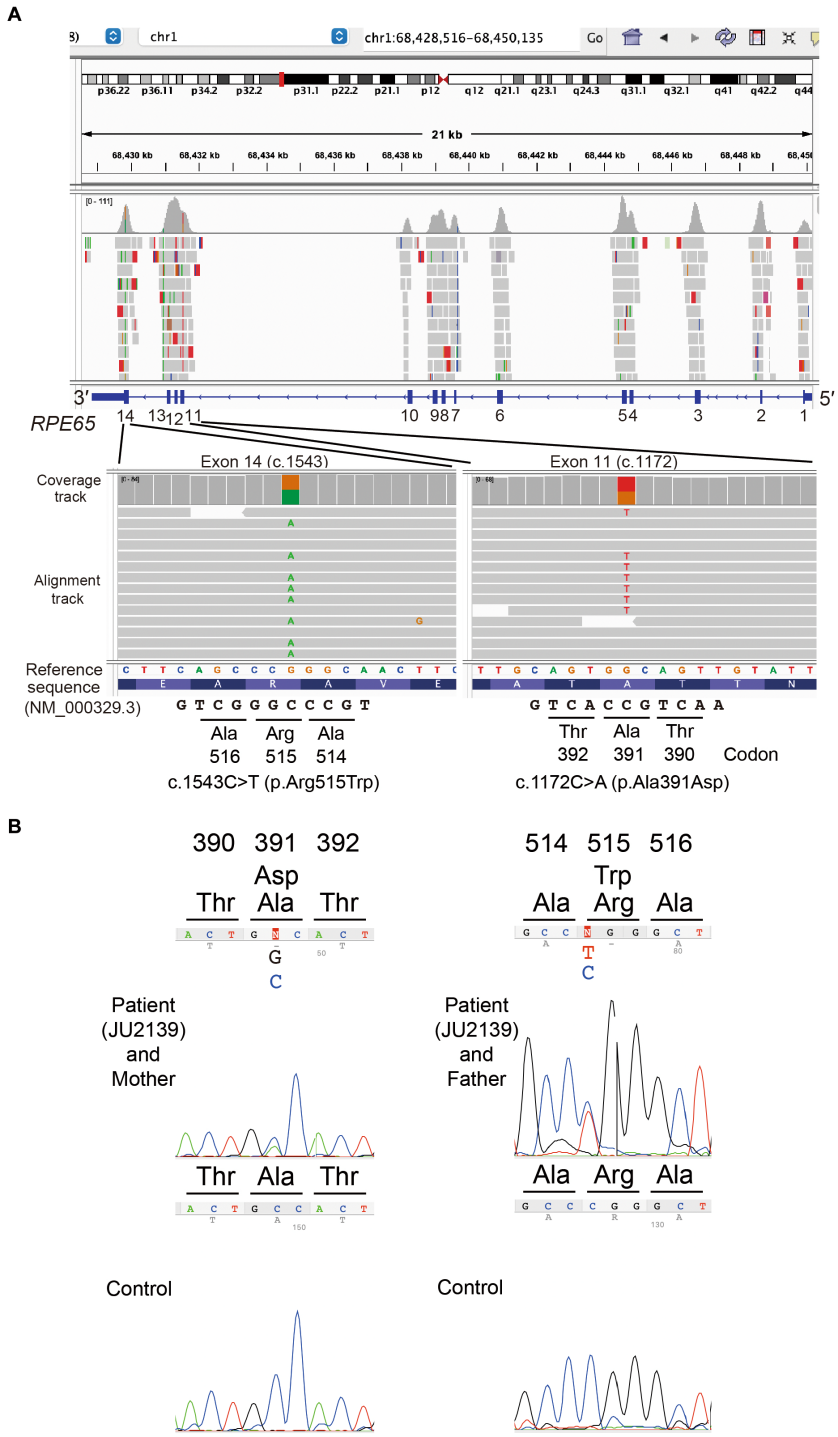
Molecular genetic analysis data. **(A)** In the patient, the Integrative Genomics Viewer screenshots of whole exome sequencing validation data show a heterozygous missense variant c.1172C > A; p.(Ala391Asp) in exon 11 and a heterozygous missense variant c.1543C > T; p.(Arg515Trp) in exon 14 of the *RPE65* gene (NM_000329.3). **(B)** Sanger sequencing chromatograms of *RPE65*. Partial nucleotide sequences demonstrate that the patient (JU2139) carries the compound heterozygous variants (c.1172C > A; p.(Ala391Asp) and c.1543C > T; p.(Arg515Trp)), whereas his mother and father carry the heterozygous variants p.(Ala391Asp) and p.(Arg515Trp), respectively.

For the purpose of genetic testing, the protocol of our study received approval from the Institutional Review Board of The Jikei University School of Medicine (approval numbers: 24-2316997). Informed consent was procured from both the patient and his parents. Genomic DNA was initially extracted from peripheral blood samples. Subsequent whole-exome sequencing (WES) and bioinformatics analyses were conducted to identify disease causing variants in the patient’s genome. The methodologies for WES have been elaborated upon in our previous publications (8, 14, 15). The Genome Reference Consortium Human Genome Build 38 (GRCh38) served as the reference sequence. Through a series of filtering steps, two missense variants in *RPE65* (NM_000329.3) were detected: c.1172C > A (chr1:68431542G > T, GRCh38.p14, rs753540419); p.(Ala391Asp) in exon 11 and c.1543C > T (chr1:68429835G > A, GRCh38.p14, rs121917745);p.(Arg515Trp) in exon 14. These variants were visualized using the Integrative Genomic Viewer (IGV version 2.16.2, Broad Institute, MA, United States) software, as depicted in Figure 3A.

Sanger sequencing was performed for validation of the variants and for cosegregation analysis. The following primer sets were used for *RPE65*: exon 11: forward primer (RPE65-11bF) 5′-GAGCCAAGACTTAAGAACTC-3′ and reverse primer (RPE65-11bR) 5′-GACTAGCATATACTCAAAGCAC-3′, and exon 14 forward primer (RPE65-14F) 5′-TTAGAGCTTACAGTGTAGGTAG-3′ and reverse primer (RPE65-14R) 5′-AAGCCATTTAGTAAGTCCAC-3′ were used. His mother carried the p.(Ala391Asp) variant heterozygously, while his father carried the p.(Arg515Trp) variant heterozygously (Figure 3B).

The p.(Ala391Asp) variant has never been previously reported, as confirmed by searches in the Human Gene Mutation Database Professional (HGMD)^1^ as of February 2023, Leiden Open Variation Database 3.0 (LOVD)^2^ and ClinVar,^3^ thus designating it as a novel missense variant. The alanine residue at position 391, within the dimer-mediating region (16, 17) composed of amino-acid residues 371-404, is highly conserved across vertebrate species. The allele frequency of the p.(Ala391Asp) variant in the Genome Aggregation Database (gnomAD)^4^ is exceedingly rare at 0.000004 (1 in 250,644 alleles). This variant is absent in the Tohoku Medical Megabank Organization’s 54 K Japanese Genome Variation Database [ToMMo 54KJPN; (18)]^5^, which includes data from 54,267 Japanese individuals. According to the American College of Medical Genetics standards, the p.(Ala391Asp) was determined to be “pathogenic” based on the following criteria: PM1 (within the dimer-mediating region), PM2 (not found in controls), PM3 (detected in trans), PP3 (computational evidence for pathogenicity), and PP4 (highly specific for the LCA phenotype). The other p.(Arg515Trp) variant, classified as pathogenic in ClinVar, has been documented in Japanese patients diagnosed with LCA (Table 1). Within the ToMMo 54KJPN, this variant’s allele frequency stands at 0.0008 (86 in 108,534 alleles), which is notably 80-fold higher than the frequency observed in gnomAD, where it is 0.00001 (4 in 280,264 alleles).

**Table 1:** Summary of Japanese patients with inherited retinal dystrophy, who carry biallelic variants in the *RPE65* gene (NM_000329.3).

Case	Patient ID	Gender	Age at onset (years)	Age at exam (years)	Diagnosis	Variant 1	Variant 2	Publication year
1	Case 1	F	Under 1 year	20	LCA	c.1349T>G:p.(L450R)	c.1543C>T:p.(R515W)	2000 (23), 2001 (21)
2	Case 2	F	Under 1 year	18	LCA	c.1349T>G:p.(L450R)	c.1543C>T:p.(R515W)	2000 (23), 2001 (21)
3	#3901	F	Early childhood	55	RP	c.1543C>T:p.(R515W)	c.1543C>T:p.(R515W)	2004 (9)
4	K1889	ND	ND	ND	RP	c.1543C>T:p.(R515W)	c.1543C>T:p.(R515W)	2014 (7)
5	K6282	ND	ND	ND	RP	c.133T>C:p.(C45R)	c.1543C>T:p.(R515W)	2014 (7)
6	MIE#0034/ME-22	F	Under 1 year	11	LCA	c.177C>G:p.(H59Q)	c.183dup:p.(D62*)	2016 (10), 2022 (8)
7	JU#0756/JK-133	F	Under 1 year	30	LCA	c.1543C>T:p.(R515W)	c.1543C>T:p.(R515W)	2016 (10), 2022 (8)
8	JU#1085	F	3	23	Early onset flecked retinal dystrophy	c.683A>C:p.(Q228P)	c.1543C>T:p.(R515W)	2018 (11)
9	JU#1303/JK-206	M	3	11	Early onset flecked retinal dystrophy	c.683A>C:p.(Q228P)	c.1028T>A:(p.L343*)	2018 (11), 2022 (8)
10	Patient ID 43	F	ND	42	RP	c.118G>A:(p.G40S)	c.177C>G:(p.H59Q)	2018 (24)
11	N-178	F	ND	41	RP	c.1543C>T:p.(R515W)	c.1543C>T:p.(R515W)	2019 (25)
12	J-13-01	F	ND	47	RP	c.1543C>T:p.(R515W)	c.1543C>T:p.(R515W)	2019 (25)
13	YWC-273	F	ND	34	RP	c.370C>T:p.(R124*)	c.1543C>T:p.(R515W)	2019 (25)
14	NG-183	F	ND	ND	RP	c.1543C>T:p.(R515W)	c.1543C>T:p.(R515W)	2019 (8)
15	TI-18	M	ND	ND	LCA	c.1543C>T:p.(R515W)	c.1543C>T:p.(R515W)	2019 (8)
16	JU#2139	M	Under 1 year	27	LCA	c.1172C>A:p.(A391D)	c.1543C>T:p.(R515W)	This report

LCA, Leber congenital amaurosis; ND, not determined; RP, retinitis pigmentosa.

## 3 Discussion

This study documents the clinical and genetic characteristics of LCA associated with biallelic *RPE65* variants in a Japanese patient, emphasizing the implications for genetic diagnosis and potential gene therapy in Japan.

Our patient exhibited early symptoms such as nystagmus and night blindness, which are typical of LCA. The progression of his visual acuity loss and the characteristic ophthalmological findings, including FAF and OCT (Figures 1B,C), are consistent with those described in *RPE65*-associated LCA/EOSRD. The absence of responses in the ERGs (Figure 2) confirms the generalized dysfunction of both rod and cone systems, indicative of LCA.

Through whole exome sequencing, we identified two missense variants in *RPE65*: the novel p.(Ala391Asp) variant and the p.(Arg515Trp) variant, which has been previously reported in Japanese patients with *RPE65*-associated LCA/EOSRD (Figures 3A,B). The novel p.(Ala391Asp) variant’s rarity is highlighted by its extremely low or non-existent allele frequency in large databases such as gnomAD and ToMMo 54KJPN (18). In contrast, the allele frequency of the recurrent p.(Arg515Trp) variant is significantly higher in the Japanese population, underscoring the importance of localized genetic databases for accurate variant interpretation and the necessity for population-specific approaches in genetic research and diagnostics.

Our report provides an opportunity to discuss the genotype–phenotype correlation, particularly in the context of the Japanese population. Although genotype–phenotype correlations in patients with *RPE65* variants are not well-defined, truncating variants appear to result in more severe clinical phenotypes (19, 20). We have compiled previous reports of *RPE65*-associated RP/EOSRD/LCA from Japan (8–11, 21–25). Including our case, only 16 cases of patients with biallelic *RPE65* variants have been documented (Table 1). Clinical diagnoses ranged from RP, EOSRD to LCA. Most patients had biallelic missense variants. Notably, the p.(Arg515Trp) variant was detected at a high frequency, accounting for 20 out of 32 alleles (62.5%) (Table 1). This aligns with allele frequency data from the Japanese database, ToMMo 54KJPN (18). When considering *RPE65*-associated LCA/EOSRD in Japanese patients, there is a high probability of encountering this frequent p.(Arg515Trp) variant. Our case also harbored the p.(Arg515Trp) variant heterozygously. Seven cases have been reported with the p.(Arg515Trp) variant in the homozygous state, with five diagnosed with RP (Case # 3, 4, 11, 12, and 14) and two with LCA (Case # 7 and 15) (Table 1). Phenotypic variability may be present even in cases with the p.(Arg515Trp) variants homozygously. On the other hand, the novel p.(Ala391Asp) variant is located within the dimer-mediating region (16, 17) of the RPE65 protein, which forms a dimer of two symmetrical enzymatically independent subunits. Therefore, the alanine at position 391 is likely to be a functionally important amino acid residue.

In 2023, Japan’s Ministry of Health, Labour and Welfare approved Voretigene neparvovec as the first gene therapy for LCA/EOSRD with biallelic *RPE65* variants in Japan. However, the lower incidence of *RPE65*-associated LCA/EOSRD in the Japanese population may influence the feasibility and prioritization of such therapies. A thorough understanding of the disease’s natural history in this demographic is crucial for initiating treatment appropriately.

This report underlines the significance of comprehensive genetic testing and detailed clinical characterization in this rare disease. It highlights potential avenues for future research into the pathophysiology of *RPE65*-associated LCA/EOSRD and emphasizes the necessity for extensive genetic studies within the Japanese population to elucidate the spectrum of *RPE65* variants and their clinical presentations.

## 4 Conclusion

This study provides valuable insights into the phenotype and genetic basis of *RPE65*-associated LCA/EOSRD in Japanese patients, establishing a foundation for improved diagnostic accuracy, informed therapeutic decisions, and future research in IRDs.

## Data Availability

The datasets for this article are not publicly available in order to protect patient anonymity. Requests to access the datasets should be directed to the corresponding author.

## Footnotes

^1^ http://www.hgmd.cf.ac.uk/

^2^ https://www.lovd.nl/3.0/home

^3^ https://www.ncbi.nlm.nih.gov/clinvar/

^4^ https://gnomad.broadinstitute.org/

^5^ https://jmorp.megabank.tohoku.ac.jp/

## References

[1] den A Roepman R Koenekoop RK Cremers FP. Leber congenital amaurosis: genes, proteins and disease mechanisms. Prog Retin Eye Res. (2008) 27:391–419. doi: 10.1016/j.preteyeres.2008.05.003 18632300

[2] Kumaran N Moore AT Weleber RG Michaelides M. Leber congenital Amaurosis/early-onset severe retinal dystrophy: clinical features, molecular genetics and therapeutic interventions. Br J Ophthalmol. (2017) 101:1147–54. doi: 10.1136/bjophthalmol-2016-309975 28689169 PMC5574398

[3] Gu SM Thompson DA Srikumari CR Lorenz B Finckh U Nicoletti A. Mutations in *Rpe65* cause autosomal recessive childhood-onset severe retinal dystrophy. Nat Genet. (1997) 17:194–7. doi: 10.1038/ng1097-194, PMID: 9326941

[4] Marlhens F Bareil C Griffoin JM Zrenner E Amalric P Eliaou C. Mutations in *RPE65* cause Leber’s congenital Amaurosis. Nat Genet. (1997) 17:139–41. doi: 10.1038/ng1097-139 9326927

[5] Thompson DA Gyurus P Fleischer LL Bingham EL McHenry CL Apfelstedt-Sylla E. Genetics and phenotypes of *RPE65* mutations in inherited retinal degeneration. Invest Ophthalmol Vis Sci. (2000) 41:4293–9. PMID: 11095629

[6] Morimura H Fishman GA Grover SA Fulton AB Berson EL Dryja TP. Mutations in the *RPE65* gene in patients with autosomal recessive retinitis Pigmentosa or Leber congenital Amaurosis. Proc Natl Acad Sci USA. (1998) 95:3088–93. doi: 10.1073/pnas.95.6.3088, PMID: 9501220 PMC19699

[7] Oishi M Oishi A Gotoh N Ogino K Higasa K Iida K. Comprehensive molecular diagnosis of a large cohort of Japanese retinitis Pigmentosa and usher syndrome patients by next-generation sequencing. Invest Ophthalmol Vis Sci. (2014) 55:7369–75. doi: 10.1167/iovs.14-15458, PMID: 25324289

[8] Suga A Yoshitake K Minematsu N Tsunoda K Fujinami K Miyake Y. Genetic characterization of 1210 Japanese pedigrees with inherited retinal diseases by whole-exome sequencing. Hum Mutat. (2022) 43:2251–64. doi: 10.1002/humu.24492 36284460

[9] Kondo H Qin M Mizota A Kondo M Hayashi H Hayashi K. A homozygosity-based search for mutations in patients with autosomal recessive retinitis Pigmentosa, using microsatellite markers. Invest Ophthalmol Vis Sci. (2004) 45:4433–9. doi: 10.1167/iovs.04-0544, PMID: 15557452

[10] Katagiri S Hayashi T Kondo M Tsukitome H Yoshitake K Akahori M. *RPE65* mutations in two Japanese families with Leber congenital Amaurosis. Ophthalmic Genet. (2016) 37:161–9. doi: 10.3109/13816810.2014.991931, PMID: 25495949

[11] Katagiri S Hosono K Hayashi T Kurata K Mizobuchi K Matsuura T. Early onset flecked retinal dystrophy associated with new compound heterozygous *RPE65* variants. Mol Vis. (2018) 24:286–96. PMID: 29681726 PMC5893010

[12] Russell S Bennett J Wellman JA Chung DC Yu ZF Tillman A. Efficacy and safety of Voretigene Neparvovec (Aav2-Hrpe65v2) in patients with *RPE65*-mediated inherited retinal dystrophy: a randomised, controlled, open-label, phase 3 trial. Lancet. (2017) 390:849–60. doi: 10.1016/S0140-6736(17)31868-8, PMID: 28712537 PMC5726391

[13] Robson AG Frishman LJ Grigg J Hamilton R Jeffrey BG Kondo M. Iscev standard for full-field clinical electroretinography (2022 update). Doc Ophthalmol. (2022) 144:165–77. doi: 10.1007/s10633-022-09872-0, PMID: 35511377 PMC9192408

[14] Hayashi T Mizobuchi K Kameya S Ueno S Matsuura T Nakano T. A mild form of *POC1B*-associated retinal dystrophy with relatively preserved cone system function. Doc Ophthalmol. (2023) 147:59–70. doi: 10.1007/s10633-023-09936-9, PMID: 37227616

[15] Morohashi T Hayashi T Mizobuchi K Nakano T Morioka I. Bardet-Biedl syndrome associated with novel compound heterozygous variants in *BBS12* gene. Doc Ophthalmol. (2023) 146:165–71. doi: 10.1007/s10633-022-09915-6, PMID: 36574078

[16] Kiser PD Farquhar ER Shi W Sui X Chance MR Palczewski K. Structure of RPE65 isomerase in a Lipidic matrix reveals roles for phospholipids and Iron in catalysis. Proc Natl Acad Sci USA. (2012) 109:E2747–56. doi: 10.1073/pnas.1212025109, PMID: 23012475 PMC3478654

[17] Poli G Barravecchia I Demontis GC Sodi A Saba A Rizzo S. Predicting potentially pathogenic effects of *h*RPE65 missense mutations: a computational strategy based on molecular dynamics simulations. J Enzyme Inhib Med Chem. (2022) 37:1765–72. doi: 10.1080/14756366.2022.2090547, PMID: 35726567 PMC9225791

[18] Tadaka S Kawashima J Hishinuma E Saito S Okamura Y Otsuki A. Jmorp: Japanese multi-omics reference panel update report 2023. Nucleic Acids Res. (2024) 52:D622–32. doi: 10.1093/nar/gkad978, PMID: 37930845 PMC10767895

[19] Gao FJ Wang DD Li JK Hu FY Xu P Chen F. Frequency and phenotypic characteristics of *RPE65* mutations in the Chinese population. Orphanet J Rare Dis. (2021) 16:174. doi: 10.1186/s13023-021-01807-3, PMID: 33952291 PMC8097799

[20] Lopez-Rodriguez R Lantero E Blanco-Kelly F Avila-Fernandez A Martin Merida I Del Pozo-Valero M. *RPE65*-related retinal dystrophy: mutational and phenotypic Spectrum in 45 affected patients. Exp Eye Res. (2021) 212:108761. doi: 10.1016/j.exer.2021.108761 34492281

[21] Wada Y Tamai M. *RPE65* gene mutations and retinal degeneration: Leber congenital Amaurosis [in Japanese]. Rinsho Ganka (Jpn J Clin Ophthalmol). (2001) 55:401–3. doi: 10.11477/mf.1410907225

[22] Wada Y Itabashi T Sukegawa M Tada A Sato H Imai E. Prevalence of mutations in seven candidate genes in Japanese patients with Leber’s congenital Amaurosis (Arvo annual meeting abstract). Invest Ophthalmol Vis Sci. (2006) 47:1691.16565410

[23] Wada Y Nakazawa T Abe T Fuse N Tamai M. Clinical Varibility of patients associated with gene mutations of visual cycle protein; Arrestin, *RPE65* and *RDH5* genes. (Arvo annual meeting abstract). Invest Ophthalmol Vis Sci. (2000) 41:S617.

[24] Maeda A Yoshida A Kawai K Arai Y Akiba R Inaba A. Development of a molecular diagnostic test for retinitis Pigmentosa in the Japanese population. Jpn J Ophthalmol. (2018) 62:451–7. doi: 10.1007/s10384-018-0601-x, PMID: 29785639

[25] Koyanagi Y Akiyama M Nishiguchi KM Momozawa Y Kamatani Y Takata S. Genetic characteristics of retinitis Pigmentosa in 1204 Japanese patients. J Med Genet. (2019) 56:662–70. doi: 10.1136/jmedgenet-2018-105691 31213501

